# Infused Therapy and Survival in Older Patients Diagnosed with Metastatic Breast Cancer who Received Trastuzumab

**DOI:** 10.3109/07357907.2011.616251

**Published:** 2011-09-19

**Authors:** Robert I Griffiths, Deepa Lalla, Robert J Herbert, Justin F Doan, Melissa G Brammer, Mark D Danese

**Affiliations:** 1 Outcomes Insights, Inc., Westlake Village, CA, USA; 2 Johns Hopkins University School of Medicine, Baltimore, MD, USA; 3 Genentech, Inc., South San Francisco, CA, USA; 4 Johns Hopkins Bloomberg School of Public Health, Johns Hopkins University, Baltimore, MD, USA

**Keywords:** Breast cancers, Chemotherapy, Outcomes research

## Abstract

We used Surveillance, Epidemiology, and End Results-Medicare data (2000-2006) to describe treatment and survival in women diagnosed with metastatic breast cancer (MBC) who received trastuzumab. There were 610 patients with a mean age of 74 years. Overall, 32% received trastuzumab alone and 47% received trastuzumab plus a taxane. In multivariate analysis, trastuzumab plus chemotherapy was associated with a lower adjusted cancer mortality rate (Hazard Ratio [HR] 0.54; 95% Confidence Interval [CI] 0.39-0.74; *p* < .001) than trastuzumab alone among patients who received trastuzumab as part of first-line therapy. Adding chemotherapy to first-line trastuzumab for metastatic breast cancer is associated with improved cancer survival.

## INTRODUCTION

In metastatic breast cancer (MBC) among patients with human epidermal growth factor receptor 2 (HER2) over-expressing disease, trastuzumab is indicated for use in combination with paclitaxel for first-line treatment, and as a single agent for those who have previously received one or more chemotherapy regimens (1). The safety and efficacy of trastuzumab in MBC have been evaluated in numerous clinical trials (2-10). Two were particularly relevant for establishing the current indication in MBC (1). One was a multicenter, randomized, open-label trial conducted in 469 women who had not been previously treated with chemotherapy for metastatic disease (1, 8). In this trial, patients were randomly assigned to receive standard chemotherapy alone (*n* = 234) or standard chemotherapy plus trastuzumab (*n* = 235). Standard chemotherapy consisted of doxorubicin (or epirubicin) and cyclophosphamide for women who had not previously received adjuvant therapy with an anthracycline, or paclitaxel for women previously treated with an anthracycline (average age 52 years, ranging from 25 to 77). Adding trastuzumab to standard chemotherapy was associated with a lower rate of death at one year (22 versus 33%, *p* = .008), a longer survival (median survival, 25.1 versus 20.3 months, *p* = .046) time, and a 20% reduction in the risk of death (8). The other study was a multicenter, open-label, single-arm clinical trial in 222 women who had relapsed following one or two prior chemotherapy regimens for metastatic disease (1, 4). In this trial, trastuzumab was studied as a single agent in a population with an average age of 50 years, ranging from 28 to 81 (4). The objective tumor response, as determined by an independent response evaluation committee, was 15% in the intent-to-treat group (4).

Although trastuzumab for MBC has been studied extensively in clinical trials, little has been published on its use in routine clinical practice, especially in populations underrepresented in the trials. Despite the fact that HER2-positive disease has a younger age distribution, older patients still comprise a significant proportion of those who might be eligible to receive trastuzumab for MBC. According to the Surveillance, Epidemiology, and End Results (SEER) program, 57% (*n* = 4,179) of the 7,331 women diagnosed with Stage IV breast cancer in 2004-2006 were aged 65 years or older (11). The objectives of this study were to describe patterns of infused therapy in a cohort of older women who first received trastuzumab following diagnosis of MBC, and to identify factors associated with longer survival.

## MATERIAL AND METHODS

### Data source

The source of data for this study was the National Cancer Institutes (NCI) SEER cancer registry linked to Medicare enrollment and claims data (SEER-Medicare data). This database has been described in detail elsewhere (12). Briefly, as of 2010, SEER collects and publishes cancer incidence and survival data from 17 population-based cancer registries throughout the United States covering approximately 26% of the US population (13). The registries routinely collect data on patient demographics, primary tumor site, tumor morphology and stage at diagnosis, first course of treatment, and follow-up for vital status. In the SEER-Medicare data, for persons age 65 years or older, 97% are eligible for Medicare, and 93% of patients in the SEER files are matched to the Medicare enrollment file (14). At the time this study was performed, the SEER-Medicare linkage included all Medicare-eligible persons from 16 of the 17 registries through 2005 and their Medicare claims for Part A (inpatient) and Part B (outpatient and physician services) through 2006.

### Patient eligibility

Patients were included in this study if they were diagnosed with MBC, defined as either (A) *de novo* Stage IV breast cancer between 2000 and 2005, or (B) *de novo* Stage 0—III breast cancer between 2000 and 2005, with a distant recurrence before the end of their Medicare claims. Distant recurrence was identified by an International Classification of Diseases, 9th Revision, Clinical Modification (ICD-9-CM) code in the medical claims for secondary cancer (197.XX-198.XX), excluding in the breast (198.81, 198.82) or in the lymph nodes (196.XX) based on algorithms for identifying cancer relapse previously reported in the literature (15, 16). These algorithms were originally developed for detecting relapse of acute myelogenous leukemia (AML), and the best among them showed a sensitivity of 86% and a specificity of 99% in this disease (15). More recently, they have been applied to, but not validated in, a study on the costs of breast cancer recurrence (16). Other inclusion criteria consisted of the following: breast cancer was the first primary cancer diagnosed; patients first received trastuzumab therapy after diagnosis of MBC; and patients were enrolled in Medicare Parts A and B, with no health maintenance organization (HMO) coverage for 12 months prior *to de novo* diagnosis of breast cancer.

Patients were excluded for any of the following reasons: male gender; trastuzumab use *prior to* diagnosis of MBC; *de novo* diagnosis of breast cancer before age of 65 years; diagnosis made by death certificate or autopsy; death within the first month following diagnosis; or Medicare enrollment less than 12 months before diagnosis.

### Treatments

For purposes of describing treatment patterns, the observation period was defined as beginning on the day MBC was diagnosed (the index date) and ending on the last day of Medicare claims (December 31, 2006) or death, whichever came first. Since, for confidentiality reasons, SEER provides only the calendar month in which cancer is diagnosed, for *de novo* Stage IV patients, the date of MBC diagnosis was defined as the first day of the calendar month in which they were diagnosed with breast cancer. For *de novo* Stage 0—III patients, the date of diagnosis was defined as the date of the first Medicare claim indicating distant recurrence. ICD-9-CM procedure codes (17) and Healthcare Common Procedure Coding System (HCPCS) codes (18) within Medicare claims were used to identify intravenous chemotherapy agents and trastuzumab administered during the observation period (19, 20).

**Figure 1:**
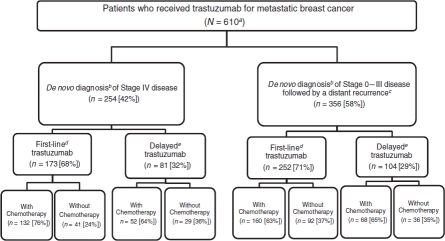
Study population. ^a^Total number of patients meeting the inclusion and exclusion criteria for the study. ^b^*De novo* staging according to SEER. ^c^ Identification of distant recurrence according to Medicare claims. ^d^Trastuzumab part of the first treatment regimen after diagnosis of metastatic breast cancer. ^e^Trastuzumab part of second or subsequent treatment after diagnosis of metastatic breast cancer.

In addition to *de novo* Stage, patients were further classified according to whether trastuzumab was part of their first infused therapy regimen during the observation period (first-line trastuzumab), or whether it was started after an initial course of chemotherapy during the observation period (delayed trastuzumab) (Figure 1). If trastuzumab began within two months of the first claim for chemotherapy, or if there was no chemotherapy claim present, the patient was classified as having received first-line trastuzumab. This regimen was then classified hierarchically using ICD-9-CM and HCPCS codes as follows: trastuzumab alone; trastuzumab plus a taxane (paclitaxel or docetaxel), with or without any other agent; and trastuzumab with any other agent except a taxane. Assignment was based on having had at least one claim for each agent during two of the first 6 months following the first chemotherapy claim.

If trastuzumab began at least 2 months after the first claim for chemotherapy, the patient was classified as having received delayed trastuzumab (Figure 1). In this group, the initial chemotherapy regimen (absent trastuzumab) was classified, using the same approach above, as: anthracycline and/or cyclophosphamide with or without any other agent; taxane and/or vinorelbine with any other agent except anthracycline or cyclophosphamide; and other. The subsequent regimen that included trastuzumab was classified the same way as the first-line trastuzumab group described above. Visual inspection of the data *prior to* the development of this classification scheme showed that in the group receiving delayed trastuzumab, there was very little overlap between the agents used in the initial chemotherapy regimen and those used in the subsequent trastuzumab regimen.

Medicare Part D (prescription drug coverage) data were not available at the time this study was performed. Consequently, it was not possible to identify anti-estrogen or other oral therapy use in this population.

### Mortality and censoring

The date of death was assigned by using the Medicare date, if available, even in cases where the SEER date also was available. The Medicare date was preferred because it is more current than the SEER date (21). In cases where the Medicare date was missing but the SEER date of death was available, the SEER date was used. All other patients were assumed to be alive at the end of the observation period (December 31, 2006), although they may have been censored earlier for other reasons, such as switching from fee-for-service to HMO coverage.

The cause of death was classified as cancer or noncancer, using the CODKM variable in the SEER Patient Entitlement and Diagnosis Summary File (PEDSF) through 2006. Cancer mortality included all deaths due to cancer (CODKM = 001-130), and not just due to breast cancer (CODKM = 046). Noncancer mortality included all other identified causes of death, e.g., CODKM =154 “Diseases of Heart” and CODKM = 148 “Diabetes Mellitus". However, it excluded missing or unspecified cause of death. These patients were censored at the time of death in both the cancer and noncancer survival analysis since exploratory analysis showed that almost 90% of those with a known cause of death died of cancer. Consequently, including them as noncancer deaths could have resulted in significant misclassification. Cancer and noncancer mortality were examined separately since the benefit of cancer therapy should be manifested primarily through differences in cancer mortality, and differences in noncancer mortality could indicate selection bias, particularly confounding by indication (22).

### Patient characteristics

Patients were described according to their demographic and clinical characteristics at the time MBC was diagnosed, with the exception of calculating the NCI Comorbidity Index (23) (described below), which was done at the time of *de novo* breast cancer diagnosis. Patient age at MBC diagnosis was stratified into five groups: 66-69; 70-74; 75-79; 80-84; and >85. Requiring eligible patients to have at least one year of Medicare enrollment *prior to* diagnosis ensured that the minimum age in the cohort was 66 years. Race/ethnicity was defined using the SEER recoded race variable as white, black, Hispanic, and other (which consists predominantly of American Indian/Native Alaskan, Native Hawaiian or Other Pacific Islander, and Asian) (24).

Medicare inpatient (Part A), outpatient, and physician (Part B) claims were used to calculate an NCI Comorbidity Index for each patient (23). This approach (25, 26) entailed first removing claims that were considered to have unreliable diagnosis coding, such as those for testing procedures used to rule out conditions. Then, remaining diagnosis and procedure codes were used to identify the 15 noncancer co-morbidities in the Charlson Comorbidity Index (CCI) (27). The algorithms used to identify these conditions reflect the Deyo (28) adaptation of the CCI, and include several procedure codes from the Romano (29) adaptation. A weight was assigned to each condition, and the weights were summed to obtain the index for each patient.

In the absence of performance status, we used Medicare claims to construct several medical resource utilization variables that have been shown and validated to predict performance status, (30) consisting of any inpatient admission (yes/no), any admission to the emergency room (yes/no), any use of durable medical equipment (yes/no), and any outpatient visit (yes/no) from 12 months before to 1 month after the diagnosis of MBC. In addition, we used SEER data and Medicare claims to identify prior surgery for breast cancer and radiation treatment (20, 31).

### Statistical analysis

Cox proportional hazards regression analysis was used to identify treatment, demographic, and clinical factors associated with overall survival. Multivariate analyses were performed on all-cause, cancer and noncancer mortality, including the entire cohort and stratified according to whether the patient received first-line or delayed trastuzumab (total of 9 analyses). We elected to conduct stratified analyses out of concern that these two groups might have significantly different prognostic features that could confound associations between treatment and survival in a single model.

In addition to the standard approach to multivariate analyses, which included individual predictors, multivariate analyses were performed using propensity techniques, (32) in which quintile of propensity score was substituted for all independent variables except initial trastuzumab regimen (total of 9 analyses), and in which the inverse of the propensity score was used as a weight in the regression analysis (total of 9 analyses).

Since treatment with trastuzumab following MBC diagnosis was a criterion for inclusion in this study, one concern in specifying this analysis was to avoid immortal time bias, (33) which, according to Suissa, is “a span of cohort follow-up during which, because of exposure definition, the outcome under study could not occur.” In this case, death could not occur between the time the patient was diagnosed with MBC and the time the patient first received trastuzumab. Therefore, the index date for this analysis was advanced from the date of diagnosis to 30 days after the date of the first claim for trastuzumab, to account also for the fact that additional time was required to determine whether or not patients received chemotherapy as part of their initial trastuzumab regimen.

## RESULTS

### Characteristics of Patients

There were 610 patients who met the study inclusion criteria (Table 1). Of these, 58% were diagnosed with Stage 0—III disease with a distant recurrence, while the remaining patients were diagnosed with *de novo* Stage IV disease. The median age at the time of MBC diagnosis was 73 years (mean 74 years), and 22% were age 80 years or older. Those who had a distant recurrence after being diagnosed with *denovo* Stage 0—III disease were significantly older *(p* < .0001). The mean time from *de novo* diagnosis of breast cancer to recurrence in this group was 17 months (median 13 months). The majority of patients were white race. However, those diagnosed with *de novo* Stage IV disease were more likely to be nonwhite *(p =* .03). Less than half of the patients were estrogen receptor (ER)—and/or progesterone receptor (PR)—positive, and differences in hormone status between the two groups were not statistically significant. Most patients had none of the co-morbidities in the NCI Comorbidity Index. Finally, almost all patients in the *de novo* Stage 0—III group had surgery for breast cancer within 4 months of diagnosis, compared with less than 50% with *de novo* Stage IV disease.

**Table 1:** Patient Characteristics

		*De Novo* Stage of Breast Cancer^a^						
			
		Stage 0-III (*n* = 356)		Stage IV (*n* = 254)		All Patients (*n* = 610)		*p* Value^b^
Age at diagnosis of metastatic breast cancer	66-69	51	14.3%	73	28.7%	124	20.3%	<.0001
	70-74	121	34.0%	74	29.1%	195	32.0%	
	75-79	88	24.7%	67	26.4%	155	25.4%	
	80-84	69	19.4%	27	10.6%	96	15.7%	
	≥85	27	7.6%	13	5.1%	40	6.6%	
Race/ethnicity	White	298	83.7%	191	75.2%	489	80.2%	.03
	Black	30	8.4%	39	15.4%	69	11.3%	
	Hispanic	16	4.5%	11	4.3%	27	4.4%	
	Other	12	3.4%	13	5.1%	25	4.1%	
Year of metastatic breast cancer diagnois	2000	16	4.5%	28	11.0%	44	7.2%	<.0001
	2001	41	11.5%	37	14.6%	78	12.8%	
	2002	46	12.9%	34	13.4%	80	13.1%	
	2003	50	14.0%	42	16.5%	92	15.1%	
	2004	69	19.4%	66	26.0%	135	22.1%	
	2005-2006	134	37.6%	47	18.5%	181	29.7%	
Estrogen(ER) and progesterone (PR) receptor status	ER+ and PR+	83	23.3%	68	26.8%	151	24.8%	.54
	ER+ or PR+	55	15.4%	42	16.5%	97	15.9%	
	ER- and PR- or unknown	218	61.2%	144	56.7%	362	59.3%	
National Cancer Institute Comorbidity Index	0	313	87.9%	226	89.0%	539	88.4%	.90
	≥1	43	12.1%	28	11.0%	71	11.6%	
Prior surgery for breast cancer		342	96.1%	124	48.8%	466	76.4%	<.0001
Radiation		192	53.9%	159	62.6%	351	57.5%	0.03
Prior inpatient admission		217	61.0%	114	44.9%	331	54.3%	<.0001
Prior emergency department Visit		37	10.4%	27	10.6%	64	10.5%	.93
Claim for durable medical equipment		141	39.6%	46	18.1%	187	30.7%	<.0001

a De *Novo* Stage could be either Stage IV or Stage 0—III. However, all patients initially diagnosed with Stage 0—III had a distant recurrence documented in their Medicare claims to be included in the study.

b All tests of significance performed using Chi Square analysis. Comparisons are *de novo* Stage IV to *de novo* Stage 0—III.

### Patterns of Trastuzumab Use

The mean time to initial trastuzumab following diagnosis of MBC was almost 6 months, with a median time of 2 months (Table 2). The median time in the *de novo* Stage 0—III with distant recurrence group was half that of the Stage IV group. However, the mean times were similar. The average duration of trastuzumab was 13 months, and it was significantly longer for those diagnosed with *de novo* Stage IV disease (16 months) compared with *de novo* Stage 0—III with distant recurrence (11 months). During trastuzumab therapy, patients received an average of 2.1 administrations per month, and there were very few calendar months during the course of therapy (6%) in which patients did not receive at least one administration of trastuzumab.

**Table 2:** Patterns of Trastuzumab use

		*De Novo* Stage of Breast Cancel^a^						
					
		Stage 0-III (*n* = 356)		Stage IV (*n* = 254)		All Patients (*n* = 610)		*p* Value^b^
Metastatic breast cancer diagnosis to first trastuzumab (days)	Mean (SD^c^)	172.8	(304.5)	176.8	(227.0)	174.5	(274.7)	.85
	Median (IQR^d^)	42	(14-173)	78	(47-189)	63	(26-183)	
Months of trastuzumab (months)	Mean (SD)	11.0	(10.5)	15.7	(14.7)	12.9	(12.6)	<.0001
	Median (IQR)	8.0	(3.0-15.0)	12.0	(4.0-22.0)	10.0	(4.0-18.0)	
Trastuzumab administrations per month	Mean (SD)	2.1	(1.0)	2.1	(1.0)	2.1	(1.0)	.30
	Median (IQR)	2	(1.1-2.9)	2.0	(1.25-3.0)	2.0	(1.16-3.0)	

a De *novo* Stage could be either Stage IV or Stage O-III. However, all patients initially diagnosed with Stage O-III had a distant recurrence documented in their Medicare claims to be included in the study.

b All tests of significance performed on means using *t-test.* Comparisons are *de novo* Stage IV to *de novo* Stage O-III.

c SD—Standard deviation.

d IQR—Interquartile range.

Overall, 70% of the cohort received first-line trastuzumab (Table 3). The proportion of patients diagnosed with *de novo* Stage IV disease was similar between patients who received first-line and those who received delayed trastuzumab. Most first-line trastuzumab patients received either monotherapy (31%) or trastuzumab plus a taxane (48%) as initial therapy following MBC diagnosis. In the delayed trastuzumab group, a slightly higher proportion of patients received trastuzumab alone (35%), and a slightly lower proportion received trastuzumab plus a taxane (45%) relative to first-line trastuzumab patients. During the observation period (2000-2006), the percent of patients receiving trastuzumab alone varied from 23 (2000) to 38% (in both 2003 and 2006).

**Table 3:** Trastuzumab Regimens

	*De Novo* Stage of Breast Cancer^a^			
			
Treatment Regimens	Stage 0-III (*n* = 356)	Stage IV (*n* = 254)	All Patients (*n* = 610)	*p* Value^b^
First-line trastuzumab (70% of cohort)	59.3 (252)	40.7 (173)	100 (425)	
Trastuzumab regimen [%(*n*)]				.02
Trastuzumab alone	36.5 (92)	23.7 (41)	31.3 (133)	
Trastuzumab plus taxane	44.4 (112)	53.2 (92)	48.0 (204)	
Trastuzumab plus other	19.1 (48)	23.1 (40)	20.7 (88)	
Delayed Trastuzumab (30% of cohort)	56.2 (104)	43.8 (81)	100 (185)	.07
Initial chemotherapy regimen [%(*n*)]				
Anthracycline and/or cyclophosphamide	41.4(43)	56.8 (46)	48.1 (89)	
Taxane and/or vinorelbine	19.2 (20)	18.5(15)	18.9 (35)	
Other (neither of the above)	39.4 (41)	24.7 (20)	33.0 (61)	
Trastuzumab Regimen [%(*n*)]				0.71
Trastuzumab alone	34.6 (36)	35.8 (29)	35.1 (65)	
Trastuzumab plus taxane	43.3 (45)	46.9 (38)	44.9 (83)	
Trastuzumab plus other	22.1 (23)	17.3 (14)	20.0 (37)	

a *De novo* Stage could be either Stage IV or Stage O-III. However, all patients initially diagnosed with Stage O-III had a distant recurrence documented in their Medicare claims to be included in the study.

b All tests of significance performed using Chi Square analysis. Comparisons are *de novo* Stage IV to *de novo* Stage O-III.

**Figure 2:**
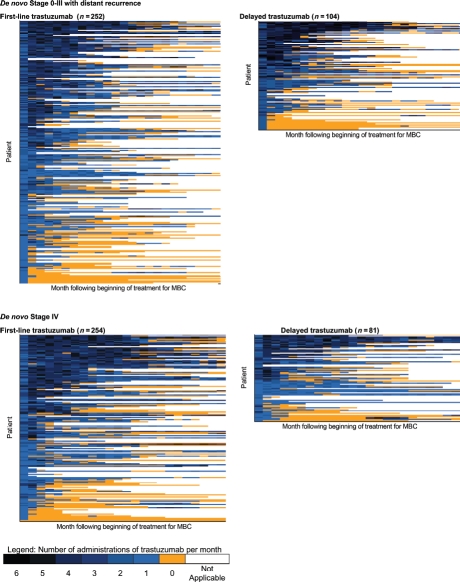
Patterns of trastuzumab use. Each row of data represents a single patient. Each colored rectangle within each row represents one month, ordered chronologically following the beginning of trastuzumab therapy, up to a maximum of 24 months. Dark blue rectangles indicate more administrations of trastuzumab in that month; light blue rectangles indicate fewer administrations; orange rectangles indicate no administrations of trastuzumab while the patient was still observed in the data set; and clear rectangles indicate that the patient was no longer observed in the data because of death or the end of the observation period. Within each of the four figures, patients are ordered from high (top of figure) to low number of trastuzumab administrations during 48 months following the beginning of therapy, a measure of both the intensity and duration of trastuzumab therapy. MBC—Metastatic breast cancer

Longitudinal “lasagna” plots (34) were constructed to illustrate the number of administrations of trastuzumab per month, the duration of trastuzumab, and gaps in administration (Figure 2) for up to 24 months following the start of treatment. These were stratified by *de novo* stage and first-line versus delayed use. In general, there was a broad spectrum of use in all four groups, ranging from uninterrupted treatment in excess of one year with four or more administrations in each month, to treatment lasting only one month with only one administration.

### Survival Analysis

Overall, 345 (57%) patients died during the observation period: 281 (81%) of these had cancer listed as the cause of death; 24 (7%) had a noncancer cause of death; and the remaining 40 (12%) had no cause of death listed and were censored in the cause-specific, but not the overall survival analysis. The estimated median survival was 566 days (95% Confidence Interval [CI] 495-644) in the entire cohort, 564 days (95% CI 473-644) in those who received first-line trastuzumab, and 579 days (95% CI 454-788) in those who received delayed trastuzumab.

In multivariate analysis that included the entire cohort, (Table 4) trastuzumab plus chemotherapy was associated with statistically significantly lower cancer mortality (Hazard Ratio [HR] 0.67; 95% CI 0.51-0.88; *p* < .01), but not non-cancer mortality.

**Table 4:** Multivariate Survival Analysis-all Patients, by Cause of Death

Patient Characteristic	All Cause Mortality 95% Confidence Interval				Cancer Mortality 95% Confidence Interval				NonCancer Mortaltiy 95% Confidence Interval				
				
		Hazard Ratio	Lower	Upper	*p* Value	Hazard Ratio	Lower	Upper	*p* Value	Hazard Ratio	Lower	Upper	*p* Value
Trastuzumab regimen included chemotherapy	No												
Without chemotherapy	Yes					Reference Category							
With chemotherapy		0.63	0.49	0.80	<.001	0.67	0.51	0.88	<.01	0.64	0.25	1.64	.35
Trastuzumab timing													
First-line						Reference Category							
Delayed		0.74	0.55	1.01	.06	0.70	0.49	0.98	.04	1.44	0.45	4.64	.54
Age at diagnosis of metastatic breast cancer	66-69					Reference Category							
	70-74	1.23	0.80	1.90	.34	1.25	0.79	1.98	.35	1.98	0.25	15.89	.52
	75-79	1.34	0.83	2.17	.23	1.30	0.77	2.21	.33	2.58	0.33	19.94	.36
	80-84	3.11	1.48	6.51	<.01	2.51	1.06	5.92	.04	1.02	0.07	15.99	.99
	≥85	5.22	1.63	16.75	<.01	3.58	1.05	12.15	.04	NA^a^	NA	NA	NA
Race	White					Reference Category							
	Black	1.51	1.08	2.12	.02	1.15	0.77	1.70	.51	4.99	1.36	18.32	.02
	Hispanic	1.10	0.57	1.83	.95	1.01	0.53	1.91	.99	1.63	0.18	14.91	.67
	Other	1.09	0.66	1.81	.74	1.20	0.67	2.17	.54	NA	NA	N/A	NA
Stage at breast cancer *de novo* diagnosis	0-III					Reference Category							
	IV	1.03	0.78	1.36	.83	0.97	0.71	1.32	.84	1.00	0.34	2.93	.99
National Cancer Institute Comorbidity Index	0					Reference Category							
	1	1.16	0.74	1.81	.52	1.12	0.68	1.85	.65	0.30	0.02	4.25	.37
	2	1.00	0.51	1.97	.10	1.05	0.50	2.23	.89	2.31	0.21	24.81	.49
		≥3	1.23	0.29	5.17	.78	0.68	0.09	5.02	.70	NA	NA	NA
Year trastuzumab therapy began	2000					Reference Category							
	2001	0.87	0.48	1.56	.63	0.95	0.49	1.83	.87	0.11	0.01	1.95	.04
	2002	1.01	0.58	1.75	.98	1.04	0.56	1.94	.90	0.84	0.16	4.43	.83
	2003	1.27	0.74	2.21	.39	1.35	0.73	2.52	.34	0.52	0.09	3.02	.46
	2004	1.03	0.60	1.77	.92	1.06	0.57	1.96	.85	0.23	0.04	1.52	.13
	2005	0.72	0.41	1.28	.26	0.78	0.41	1.48	.45	0.36	0.06	2.31	.28
	2006	0.65	0.30	1.40	.27	0.78	0.34	1.77	.55	0.40	0.03	5.92	.50
Estrogen (ER) and progesterone (PR) receptor status	ER-/PR-					Reference Category							
	ER+/PR+	0.82	0.62	1.08	.15	0.78	0.58	1.06	.11	1.17	0.39	3.47	.78
	ER+/PR-	0.69	0.50	0.96	.03	0.74	0.52	1.06	.10	0.42	0.08	2.13	.29
	ER-/PR+	0.82	0.38	1.79	.62	0.73	0.30	1.80	.49	4.46	0.37	54.01	.24
Prior surgery for breast cancer	No					Reference Category							
	Yes	0.78	0.58	1.05	.10	0.67	0.49	0.94	.02	0.81	0.20	3.27	.77
Radiation	No					Reference Category							
	Yes	1.04	0.82	1.31	.77	1.08	0.83	1.40	.59	0.42	0.15	1.15	.09
Inpatient admission	No					Reference Category							
	Yes	1.54	1.20	1.97	<.01	1.53	1.16	2.01	<.01	2.13	0.76	5.95	.15
Durable medical equipment	No					Reference Category							
	Yes	0.90	0.70	1.17	.44	0.90	0.67	1.19	.45	0.68	0.22	2.10	.50
Emergency department visit	No					Reference Category							
	Yes	1.00	0.70	1.45	.99	1.03	0.69	1.53	.91	0.19	0.02	2.07	.17
Outpatient visit	No					Reference Category							
	Yes	3.52	1.24	9.95	.02	2.64	0.92	7.55	0.07	NA	NA	NA	NA

a Not applicable-insufficient patients in this group to calculate estimate.

**Figure 3:**
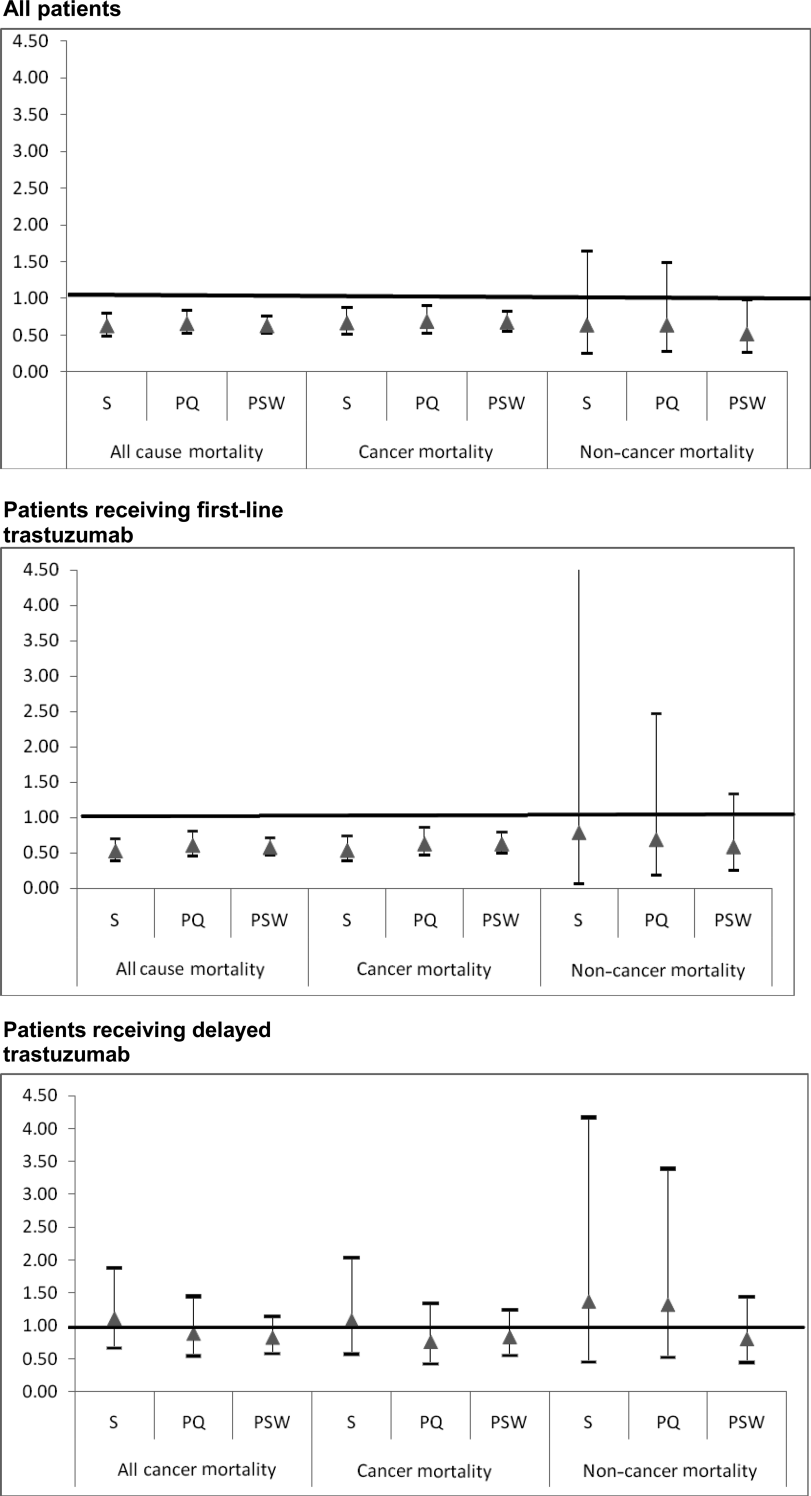
Sensitivity of trastuzumab hazard ratio to changes in the multivariate survival analysis. This figure presents the results of three sets (all patients, patients receiving first-line trastuzumab, patients receiving delayed trastuzumab) of nine multivariate survival analyses (three each for all-cause, cancer, and noncancer mortality) designed to test the sensitivity of the findings reported in Tables 4 and 5 to changes in the approach to multivariate analysis. Standard multivariate survival analyses (S) were performed with all individual patient variables included in the model. Propensity multivariate survival analyses were performed with either propensity score quintile (PQ) included in the model as a substitute for all patient variables except trastuzumab plus chemotherapy, or using propensity score as a weight (PSW). The y-axis indicates the hazard ratio for trastuzumab plus chemotherapy compared with trastuzumab alone. Triangles represent the estimated hazard ratio for trastuzumab plus chemotherapy compared with trastuzumab alone from the corresponding model on the x-axis. Bars around each triangle represent the upper and lower bounds of the 95% confidence interval for the hazard ratio. Confidence intervals that overlap the horizontal line at the hazard ratio of 1.0 indicate that the estimated hazard ratio for trastuzumab plus chemotherapy is not significant at p = .05.

In stratified analysis, (Table 5) first-line trastuzumab plus chemotherapy was associated with statistically significantly lower cancer mortality (HR 0.54; 95% CI 0.39-0.74; *p <* .001). However, adding chemotherapy to delayed trastuzumab had no impact on cancer mortality. Findings from the propensity score analysis were consistent with those from the standard regression analysis (Figure 3).

**Table 5:** Multivariate Survival Analysis, Cancer Mortality Stratified by First-line Versus Delayed Trastuzumab

		First-Line Trastuzumab				Delayed Trastuzumab			
			
			95% Confidence Interval				95% Confidence Interval		
							
Patient Characteristic		Hazard Ratio	Lower	Upper	*p* value	Hazard Ratio	Lower	Upper	*p* value
Trastuzumab regimen included chemotherapy	No				Reference Category				
		Yes	0.54	0.39	0.74 <.001	1.44	0.77	2.69	.25
Age at diagnosis of metastatic breast cancer	66-69				Reference Category				
	70-74	1.28	0.71	2.34	.41	1.04	0.42	2.57	.93
	75-79	1.10	0.59	2.02	.77	1.58	0.40	6.28	.52
	≥85	4.54	1.18	17.53	.03	NA^a^	NA	NA	NA
Race/ethnicity	White				Reference Category				
	Black	1.18	0.72	1.93	.51	0.95	0.41	2.22	.90
	Hispanic	1.12	0.59	2.12	.72	0.54	0.12	2.49	.43
	Other	1.05	0.40	2.72	.92	1.18	0.42	3.37	.75
Stage at breast cancer *de novo* diagnosis	0-III				Reference Category				
	IV	0.91	0.61	1.37	.66	1.26	0.65	2.45	.49
National Cancer Institute Comorbidity Index	0				Reference Category				
	1	1.38	0.76	2.51	.29	0.44	0.13	1.51	.19
	2	1.03	0.45	2.35	.94	1.52	0.17	13.83	.71
	≥3	0.59	0.08	4.45	.61	NA	NA	NA	NA
Year trastuzumab therapy began	2000				Reference Category				
	2001	1.24	0.58	2.67	.58	0.53	0.11	2.52	.42
	2002	1.20	0.58	2.47	.62	1.10	0.25	4.79	.90
	2003	1.49	0.72	3.09	.28	1.31	0.31	5.59	.71
	2004	1.19	0.58	2.44	.64	1.18	0.27	5.11	.83
	2005	0.85	0.40	1.78	.66	0.54	0.12	2.40	.42
	2006	1.14	0.45	2.87	.78	0.17	0.01	1.93	.15
Estrogen (ER) and Progesterone (PR) receptor status	ER∼/PR∼				Reference Category				
	ER+/PR+	0.59	0.41	0.86	.01	1.68	0.88	3.21	.12
	ER+/PR-	0.64	0.41	0.99	.04	0.61	0.28	1.32	.21
	ER-/PR+	0.58	0.20	1.66	.31	1.13	0.08	16.29	.93
Prior surgery for breast cancer	No				Reference Category				
	Yes	0.74	0.50	1.12	.15	0.34	0.17	0.70	<.01
Radiation	No				Reference Category				
	Yes	0.97	0.72	1.31	.84	1.54	0.78	3.02	.21
Inpatient admission	No				Reference Category				
	Yes	1.37	0.97	1.93	.07	1.59	0.94	2.69	.09
Emergency department visit	No				Reference Category				
	Yes	1.24	0.77	1.20	.38	1.24	0.47	3.32	.66
Outpatient visit	No				Reference Category				
	Yes	2.43	0.69	8.60	.17	5.57	0.63	49.02	.12
Durable medical equipment	No				Reference Category				
	Yes	0.91	0.66	1.27	.59	1.11	0.54	2.27	.78

a Not applicable-insufficient patients in this group to calculate estimate.

## DISCUSSION

We conducted a study using SEER-Medicare to identify patterns of infused therapy and survival in a cohort of older women who received trastuzumab therapy for MBC. Our findings show that following diagnosis of MBC, 70% of the cohort received first-line trastuzumab therapy, while the remainder received delayed trastuzumab. As first-line treatment in MBC, trastuzumab is indicated for use in combination with paclitaxel (1). However, we found that almost one-third of these older patients who received first-line trastuzumab received it without infused chemotherapy. The use of trastuzumab monotherapy first-line was more common in patients diagnosed with *de novo* Stage 0—III disease and a distant recurrence, compared with *de novo* Stage IV disease (37% versus 24%). This suggests that the decision to select trastuzumab monotherapy versus trastuzumab in combination with one or more chemotherapy agents may have been influenced by prior use of chemotherapy in the adjuvant setting. Also, since this study was conducted in patients diagnosed with breast cancer from 2000-2005, the discrepancy between the labeled indication and what we observed may well reflect the impact of two important clinical trials of first-line trastuzumab monotherapy for MBC, (5, 7) both of which were published several years after trastuzumab was approved by the Food and Drug Administration (FDA).

Overall, the results of the multivariate analysis show that adding chemotherapy to trastuzumab improves survival in patients with MBC. More detailed examination indicates that this is true only for those who received trastuzumab as first-line therapy and for cancer mortality alone. The findings were robust to several different analytic approaches, including propensity techniques.

The finding that adding chemotherapy to first-line trastuzumab was associated with improved survival relative to trastuzumab alone raises important questions with regard to the optimal treatment regimen in these patients. According to one recent review, (2) at the time the patients in our study were treated there had not been a randomized study of trastuzumab with or without chemotherapy. However, a recently published randomized trial of trastuzumab monotherapy versus trastuzumab plus docetaxel as first-line therapy in MBC, (10) was stopped early after an interim analysis showed a significantly lower mortality rate in the patients receiving chemotherapy with trastuzumab (HR for survival 2.72 *p* = .04). Upon inverting the HR for survival, we find that the HR for overall mortality (0.37) in this trial was similar to the HR ratio for cancer mortality among patients receiving first-line trastuzumab plus chemotherapy (0.54) in our study. Therefore, our findings based on data from routine clinical practice appear to support the findings from this recent trial, and suggest they apply to elderly patients.

The main limitation of this study, as with any observational study that compares the effects of alternative interventions, is the possibility that patients were selected into the treatment groups for reasons related to survival, (22, 35, 36) and that we failed to account for these reasons in our analyses. Although no approach completely eliminates confounding by indication in observational studies, (36) we took several steps to minimize its impact here. One possibility is that patients with poorer performance status or more comorbidity were selected to receive trastuzumab monotherapy due to concerns about the toxicity of chemotherapy. (22) SEER-Medicare does not include performance status. Consequently, using Medicare claims we constructed several medical resource use variables that have been validated to predict ECOG and Karnofsky performance status (30). Also, we examined cancer and noncancer mortality based on the rationale that any real benefit of cancer treatment should be observed only through cancer-specific mortality, and that differences in noncancer mortality suggest confounding by indication (22). In this study, we found adding chemotherapy to trastuzumab was associated with a significantly lower rate of cancer but not noncancer mortality.

Although the coefficients for trastuzumab plus chemotherapy were similar for cancer and noncancer mortality, it is important to note that only 24 patients (8% of those with a known cause of death) had a noncancer cause of death. Consequently, the coefficients in these models are unstable and their direction and significance can be determined by a few individuals. Finally, we used two different approaches to multivariate survival analysis: the standard approach in which all patient characteristics were included as independent variables, and propensity techniques (32) in which propensity score quintile was substituted for the vector of independent variables except treatment, and alternatively where the raw propensity score was used as a weight. It should be noted, however, that propensity techniques do not account for unobserved confounding covariates (32). Instrumental variable approaches have been applied to SEER-Medicare analysis, but to our knowledge not in small cohorts such as ours.

A second limitation of this study is that we did not include a comparator group of patients who did not receive trastuzumab. The main reason is that, presently, SEER-Medicare does not include information on HER2 status. It can be inferred that any patient who received trastuzumab had HER2 overexpressing disease, based on the fact that Medicare does not pay for trastuzumab without a positive HER2 test result. However, it is not possible to identify patients who are HER2 positive but who do not receive trastuzumab. Since HER2 overexpression is a significant predictor of both overall survival and time to relapse in patients with breast cancer, (37) the absence of HER2 test results is a significant barrier to conducting comparative effectiveness research (e.g. chemotherapy with versus without trastuzumab) on trastuzumab using this database, because one cannot identify a HER2 positive control group. Second, while trastuzumab was first approved by FDA in September 1998, (38) a Medicare reimbursement code specifically for trastuzumab did not become effective until January 1, 2000. Therefore, we were unable to document the use of trastuzumab during the first year following approval.

Third, as illustrated, patterns of trastuzumab use were diverse, ranging from long, uninterrupted periods with multiple administrations per month to short periods with only one administration per month. While this diversity makes it difficult to assess the outcomes effects of trastuzumab under “optimal use,” our analysis does reflect its impact in routine clinical practice.

Fourth we used an algorithm based on Medicare claims (15, 16) to identify distant recurrence (metastatic disease) among those patients who were initially diagnosed with Stage 0—III disease. Although claims-based algorithms for identifying relapse/recurrence have been validated in AML, (15) they have not been validated in breast cancer. One study conducted before the development of the algorithms for AML found that Medicare claims within 3 months of the SEER date of breast cancer diagnosis correctly identified only 60% of those with distant Stage disease at diagnosis according to SEER (39). One important difference, however, is that we used claims to identify recurrence in those previously diagnosed and staged using SEER data.

Nevertheless, to gain additional insight on this issue, we applied this algorithm to a much larger cohort of women in SEER-Medicare who were diagnosed with early-Stage (stage I—III) breast cancer, and who received adjuvant chemotherapy. In this cohort, the cumulative rate of recurrence we observed, approximately 30% after 4 years, was similar to that for anthracycline- and CMF- (cyclophosphamide, methotrexate, fluorouracil) based regimens for early breast cancer in older women as reported by the Early Breast Cancer Trialists’ Collaborative Group (EBCTCG) (40). Also, the distribution of sites of distant recurrence was consistent with what has been previously reported.

Finally, we did not investigate the use of trastuzumab in the adjuvant setting since it was not approved for this indication until 2006.

Despite the aforementioned limitations, to our knowledge, this is the first study to describe the use of trastuzumab for MBC in a large cohort of older women diagnosed with breast cancer. It shows treatment patterns consistent with the literature published around the time treatment decisions within the cohort were being made, but less consistent with the product labeling. Also, aside from one recent trial discussed above (10), we are unaware of any other study, observational or experimental, comparing survival in patients who receive trastuzumab alone versus in combination with chemotherapy for MBC. Our findings suggest trastuzumab in combination with chemotherapy is more effective than trastuzumab alone when used as first-line therapy for MBC. Since almost one-third of the first-line trastuzumab patients in our cohort received trastuzumab alone, a potential area for further research would be investigating the reasons clinicians and patients might elect to use trastuzumab monotherapy instead of trastuzumab plus chemotherapy.

## References

[1] Genentech, Inc (2009). Prescribing Information for Herceptin (trastuzumab). http://www.gene.com/gene/products/information/pdf/herceptin-prescribing.pdf.

[2] Hudis CA (2007). Trastuzumab—Mechanism of action and use in clinical practice. N Engl J Med.

[3] Baselga J, Tripathy D, Mendelsohn J, Baughman S, Benz CC, Dantis L, Sklarin NT, Seidman AD, Hudis CA, Moore J, Rosen PP, Twaddell T, Henderson IC, Norton L (1996). Phase II study of weekly intravenous recombinant humanized anti-p185HER2 monoclonal antibody in patients with HER2/neu-overexpressing metastatic breast cancer. J Clin Oncol.

[4] Cobleigh MA, Vogel CL, Tripathy D, Nicholas RJ, Scholl S, Fehrenbacher L, Wolter JM, Paton V, Shak S, Lieberman G, Slamon DJ (1999). Multinational study of the efficacy and safety of humanized anti-HER2 monoclonal antibody in women who have HER2-overexpressing metastatic breast cancer that has progressed after chemotherapy for metastatic disease. J Clin Oncol.

[5] Vogel CL, Cobleigh MA, Tripathy D, Gutheil JC, Harris LN, Fehrenbacher L, Slamon DJ, Murphy M, Novotny WF, Burchmore M, Shak S, Stewart SJ, Press M (2002). Efficacy and safety of trastuzumab as a single agent in first-line treatment of HER2-overexpressing metastatic breast cancer. J Clin Oncol.

[6] Leyland-Jones B, Gelman K, Ayoub J-P, Arnold A, Verma S, Dias R, Ghahramani P (2003). Pharmacokinetics, safety, and efficacy of trastuzumab administered every three weeks in combination with paclitaxel. J Clin Oncol.

[7] Baselga J, Carbonell X, Castaneda-Soto N-J, Clemens M, Green M, Harvey V, Morales S, Barton C, Ghahramani P (2005). Phase II study of efficacy, safety, and pharmacokinetics of trastuzumab monotherapy administered on a 3-weekly schedule. J Clin Oncol.

[8] Slamon DJ, Leyland-Jones B, Shak S, Fuchs H, Paton V, Bajamonde A, Fleming T, Eiermann W, Wolter J, Pegram M, Baselga J, Norton L (2001). Use of chemotherapy plus a monoclonal antibody against HER2 for metastatic breast cancer that overexpresses HER2. N Engl J Med.

[9] Marty M, Cognetti F, Maraninchi D, Snyder R, Mauriac L, Tubiana-Hulin M, Chan S, Grimes D, Anton A, Lluch A, Kennedy J, O'Byrne K, Conte P, Green M, Ward C, Mayne K, Extra J-M (2005). Randomized phase II trial of the efficacy and safety of trastuzumab combined with docetaxel in patients with human epidermal growth factor receptor 2—positive metastatic breast cancer administered as first-line treatment: the M77001 study group. J Clin Oncol.

[10] Inoue K, Nakagami K, Mizutani M, Hozumi Y, Fujiwara Y, Masuda N, Tsukamoto F, Saito M, Miura S, Eguchi K, Shinkai T, Ando M, Watanabe T, Masuda N, Ohashi Y, Sano M, Noguchi S (2010). Randomized phase III trial of trastuzumab monotherapy followed by trastuzumab plus docetaxel versus trastuzumab plus docetaxel as first-line therapy in patients with HER2-positive metastatic breast cancer: the J017360 trial group. Breast Cancer Res Treat.

[11] http://www.seer.cancer.gov.

[12] Warren JL, Klabunde CN, Schrag D, Bach PB, Riley GF (2002). Overview of SEER-Medicare data: content, research applications, and generalizability to the United States elderly population. Medical Care.

[13] National Cancer Institute (NCI) (2010). Overview of the SEER program [Internet]. http://seer.cancer.gov/about.

[14] National Cancer Institute (NCI) (2010). SEER-Medicare: How the SEER & Medicare data are Linked [Internet]. http://healthservices.cancer.gov/seermedicare/overview/linked.html.

[15] Earle CC, Nattinger AB, Potosky AL, Lang K, Mallick R, Berger M, Warren JL (2002). Identifying cancer relapse using SEER-Medicare Data. Medical Care.

[16] Stokes ME, Thompson D, Montoya EL, Weinstein MC, Winder EP, Earle CC (2008). Ten-year survival and cost following breast cancer recurrence: estimates from SEER-Medicare data. Value in Health.

[17] Practice Management Information Corporation (PMIC) (2005). ICD-9-CM.

[18] Practice Management Information Corporation (2005). HCPCS.

[19] Warren JL, Harlan LC, Fahey BA, Freeman JL, Klabunde CN, Cooper GS, Knopf KB (2002). Utility of the SEER-Medicare data to identify chemotherapy use. Medical Care.

[20] National Cancer Institute (NCI) (2010). Procedure codes for SEER-Medicare analysis [internet]. http://healthservices.cancer.gov/seermedicare/considerations/procedure-codes.html.

[21] ResDAC Research Data Assistance Center CMS 301: Using SEER/Medicare data for research.

[22] Giordano SH, Kuo Y-F, Duan Z, Hortobagyi GN, Freeman J, Goodwin JS (2008). Limits of observational data in dertermining out-comes from cancer therapy. Cancer.

[23] Klabunde CN, Potosky AL, Legler JM, Warren JL (2000). Development of a comorbidity index using physician claims data. J Clin Epidemiol.

[24] Fritz A, Ries L (1998). SEER Program Code Manual. http://seer.cancer.gov/manuals/codeman.pdf.

[25] National Cancer Institute (NCI) SAS macro to remove unreliable diagnosis coding. http://healthservices.cancer.gov/seermedicare/program/remove.ruleout.dxcodes.macro.txt.

[26] National Cancer Institute (NCI) SAS macro to calculate a comorbidity index for a patient with respect to cancer. NCI.

[27] Charlson ME, Pompei P, Ales KL, MacKenzie CR (1987). A new method of classifying prognostic comorbidity in longitudinal studies: development and validation. J Chron Dis.

[28] Deyo RA, Cherkin DC, Ciol MA (1992). Adapting a clinical comorbidity index for use with ICD-9-CM administrative databases. J Clin Epidemiol.

[29] Romano PS, Roos LL, Luft HS, Jollis JG, Doliszny K (1994). A comparison of administrative versus clinical data: coronary artery bypass surgery as an example. J Clin Epidemiol.

[30] Salloum RG, Smith TJ, Jensen GA, Elston Lafata J (2010). Using claims-based measures to predict performance status score in patients with lung cancer. Cancer.

[31] Virnig BA, Warren JL, Cooper GS, Klabunde CN, Schussler N, Freeman J (2002). Studying radiation therapy using SEER-Medicare-linked data. Medical Care.

[32] Rubin DB (1997). Estimating causal effects from large data sets using propensity scores. Ann Intern Med.

[33] Suissa S (2008). Immortal time bias in pharmacoepidemiology. Am J Epidemiol.

[34] Swihart BJ, Caffo B, James BD, Strand M, Schwartz BS, Punjabi NM (2010). Lasagna plots: a saucy alternative to spagetti plots. Epidemiology.

[35] Hadley J, Yabroff KR, Barrett MJ, Penson DF, Saigal CS, Potosky AL (2010). Comparative effectiveness of prostate cancer treatments: evaluating statistical adjustments for confounding in observational data. J Natl Cancer Inst.

[36] Bosco JLF, Silliman RA, Thwin SS, Geiger AM, Buist DS, Prout MN, Yood MU, Haque R, Wei F, Lash TL (2010). A most stubborn bias: no adjustment method fully resolves confounding by indication in observational studies. J Clin Epidemiol.

[37] Slamon DJ, Clark GM, Wong SG, Levin WJ, Ullrich A, McGuire WL (1987). Human breast cancer: correlation of relapse and survival with amplification of the HER-2/neu oncogene. Science.

[38] Siegel J (1998). Approval of biologies license application for Trastuzumab. http://www.fda.gov/downloads/Drugs/DevelopmentApprovalProcess/HowDrugsareDevelopedandApproved/ApprovalApplications/TherapeuticBiologicApplications/ucm091360.pdf.

[39] Cooper GS, Yuan Z, Stange KC, Amini SB, Dennis LK, Rimm AA (1999). The utility of Medicare claims data for measuring cancer stage. Medical Care.

[40] Early Breast Cancer Trialists’ Collaborative Group (EBCTCG) (2005). Effects of chemotherapy and hormonal therapy for early breast cancer on recurrence and 15-year survival: an overview of the randomized trials. Lancet.

